# Reliability of computed tomography-based renal cortex volume to determine split renal function in preoperative living kidney donors

**DOI:** 10.1259/bjro.20190025

**Published:** 2019-08-12

**Authors:** Chuthaporn Surawech, Kewalee Sasiwimonphan

**Affiliations:** Department of Radiology, Division of Diagnostic Radiology, Faculty of Medicine, Chulalongkorn University, Bangkok, Thailand

## Abstract

**Objective:**

The purpose of this study was to assess the utility of CT-based renal cortex volume to estimate split renal function (SRF) in pre-transplant living kidney donors and to evaluate its reliability to predict graft function in the recipients.

**Methods:**

Our study recruited all adult potential donors who had both Tc-99m-diethylenetriamine pentacetate (DTPA) scintigraphy and CT angiography of the abdominal aorta done before donating their kidney. We compared the correlation between CT-based renal cortex volume combined with kidney function and DTPA scan as well as post-donation kidney function in the recipients.

**Results:**

The correlation between CT-based split cortex volume and DTPA-measured SRF before transplantation was strong (intraclass correlation coefficient = 0.954–0.968). The inter-rater reliability of two radiologists also showed substantial agreement (intraclass correlation coefficient = 0.97, *p* < 0.001). In contrast, the correlations between renal cortical volume of donated kidney adjusted to recipient body weight and recipient kidney function was poor at both 2 week and 2 year follow-up.

**Conclusion:**

CT-based renal cortex volume combined with pre-operative kidney function appears to be precise and reproducible to evaluate pre-transplant SRF. Nevertheless, the prediction of recipient graft function needs to be further investigated to ensure a good outcome.

**Advances in knowledge:**

This method is practicable for all potential donors who undergo kidney transplantation in terms of streamline donor workup without compromising information.

## Introduction

Kidney transplantation from living kidney donors (LKDs) is the treatment of choice for many patients with end stage renal disease (ESRD). Living kidney transplantation has a better long-term outcome compared to cadaveric donor and the life expectancy of the donors is comparable to the general population; there is no increased risk of ESRD in the donors after having donated their kidney.^1^ According to the 2015 Annual report of organ transplantation in Thailand, there is an increase in kidney transplantation; the number of both living and deceased kidney transplantations in 2014 were approximately 5.4% (from 222 to 234) and 11.6% (from 329 to 367), respectively.^2^

Evaluation of living donor’s kidney function is necessary to ensure that the donors will have sufficient residual kidney function to live their life without any complications. Currently, there is no standard to estimate the renal function in pre-transplant LKDs.^3^ Aside from that, it is also important that the recipient’s renal function must be adequate following kidney transplantation.

According to the King Chulalongkorn Memorial hospital’s (KCMH) guideline, the renal function of the donor must be investigated before kidney transplantation by using the endogenous 24 h urine creatinine clearance (CCr) and estimated glomerular filtration rate (eGFR) as per the chronic kidney disease epidemiology (CKD-EPI) 2009 formula or Thai eGFR. CT angiography (CTA) of the abdominal aorta is used to evaluate the anatomy and vascular structures of the kidney. Tc-99m-diethylenetriaminepentacetate (DTPA) scintigraphy is used to estimate differential or split renal function (SRF). This calculation is universally performed and notably useful because eGFR and serum creatinine may not be able to determine unilateral function. Normally, the relative contribution for each kidney is between 45 and 55%.^4^ If there is asymmetry in GFR, the kidney with lesser function should be chosen for donation.

Since the nephrons’ activities in the kidney cannot be measured directly, hence the GFR measurement was used; GFR is equivalent to the total filtration rates of the functioning nephrons in the kidney. The gold-standard for GFR measurement is the assessment of urinary or plasma clearance of inulin, iothalamate or iohexol. To simplify this procedure, many alternative methods and markers have been used to estimate GFR instead such as CCr and serum Cr.^5^

In theory, an ideal glomerular filtration agent should be freely filtered through the glomeruli and not bound by plasma protein as well as not reabsorbed or secreted in the tubuli. The intravenous contrast medium used in CT examinations also has these properties; therefore, accumulation of the contrast medium is proportional to the GFR of that kidney.^6,7^

Several previous studies developed many CT volumetry techniques for calculating split renal function and it was concluded that CT could replace renal scintigraphy.^8–11^ In June 2016, Wahba et al analyzed living kidney volume by using three different volumetry techniques (*e.g.* modified ellipsoid volume, smart region of interest volumetry and renal cortex volumetry) and found that the renal cortex volumetry was the most accurate technique to evaluate pre-donation SRF.^11^ Furthermore, the advantages of CT volumetry are non-invasive, easy to perform, less time-consuming and can avoid unnecessary radiation exposure (approximately 1.5–2 mSv) from additional renal scintigraphy.^4^ The aim of this study was to assess the utility of CT-based renal cortex volume to estimate the split renal function in pre-transplant LKDs in KCMH and to evaluate its ability to predict the graft function in the recipients.

## methods and materials

### Patients

The study was approved by the institutional review board before any of the procedures were carried out. We retrospectively included all adult potential donors who underwent kidney transplantation at the KCMH between January 2012 and December 2016. Data were extracted from the database of Kidney Transplantation unit, Division of Nephrology, Department of Medicine, KCMH.

A total of 109 patients were identified. 11 patients were excluded because there were no images available in the picture archiving and communication system.

Age, sex, nephrectomy side, interval between CTA of the abdominal aorta and DTPA scintigraphy studies as well as pre-operative general renal function (*e.g.,* serum Cr, CCr and Thai eGFR) and DTPA scintigraphy were recorded for every donor. The kidney function in the recipients at 2 weeks and 2 years after transplantation were collected.

### MDCT protocol

CT examinations were performed on MDCT scanners (Siemens Somatom Force and Toshiba Aquilion One; acquisition of 192 × 0.6 mm and 64 × 0.5 mm collimation, respectively) with slice thickness/increment of 2.0/1.0 mm and 120-kV tube potential. Tube current modulation was applied. Pre- and post-contrast CT images (plain, corticomedullary, nephrographic and excretory phases) were obtained before and after intravenous administration of 100 ml of iodinated contrast agent (Omnipaque 350 mg ml^−1^). The contrast injection rate was 5 ml s^−1^. The corticomedullary acquisition used bolus tracking with region of interest (ROI) placed within the lumen of the descending aorta and had a trigger threshold of 330 Hounsfield units (HU). For nephrographic and excretory phases, a posttrigger threshold delay of 90 s and 10 min were used. Sagittal and coronal maximal intensity projections (MIP) were reconstructed with slice thickness/increment of 5.0/3.0 mm.

In our study, the contrast-to-noise ratio (CNR) was used to monitor image quality of the evaluated scans, which were derived from the differences of HU between cortex and the medulla divided by the standard deviation (SD) of the medulla. The image quality is justifiable if the CNR is greater than 5.

### Image analysis

**Figure 1.**
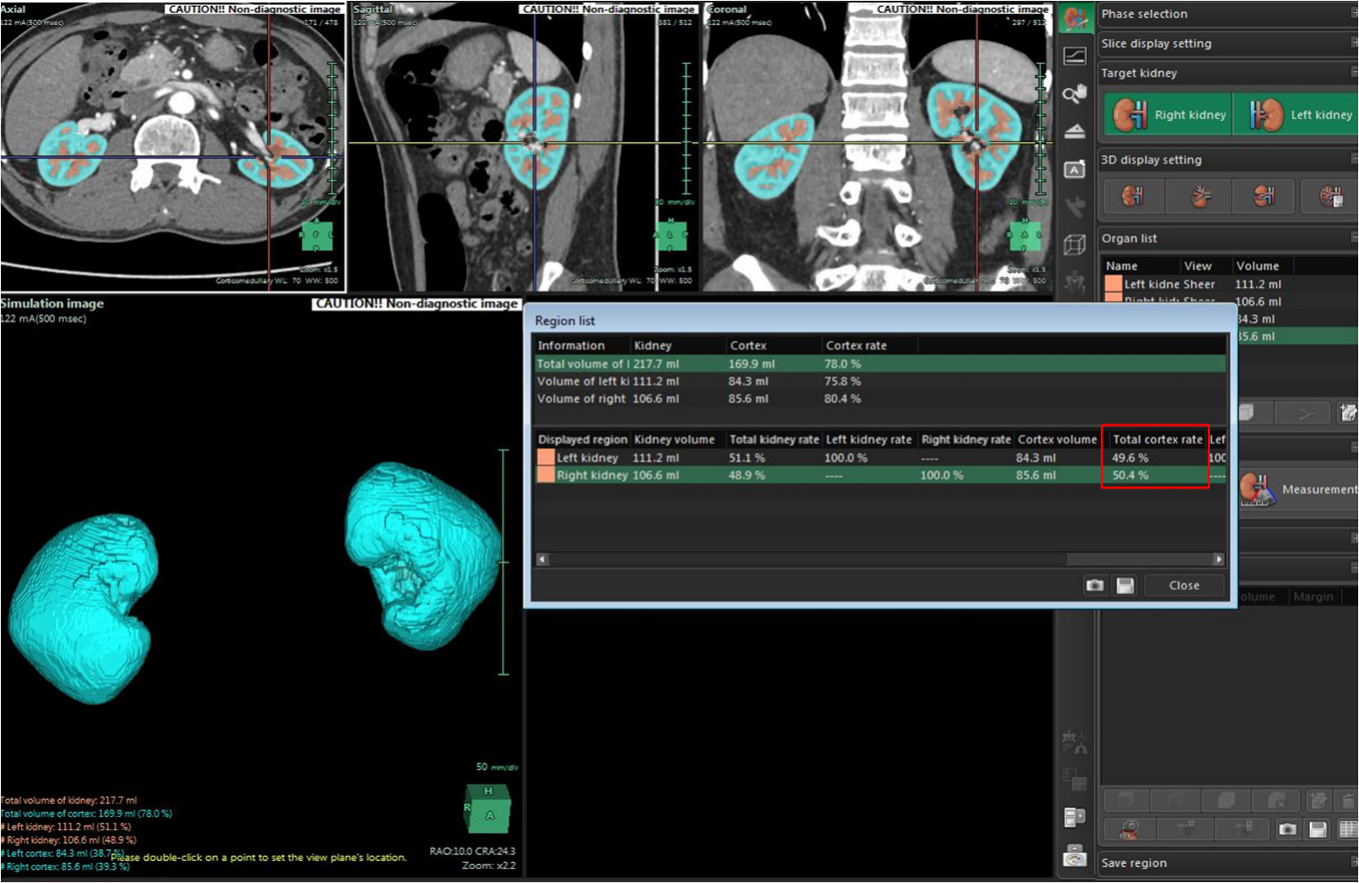
The software estimated the RCV on corticomedullary CT images. RCV, renal cortical volume.

Two independent radiologists used the image-recognition or SYNAPSE 3D software (Synapse Vincent System, v. 4; Fujifilm Corporation, Tokyo, Japan) to automatically delineate each renal cortex of the donor on corticomedullary CT images based on HU threshold, and then the software estimated the volume, separately. If there was an error (*e.g.* the program improperly shaped the kidney), the kidney contour was then drawn manually, slice by slice instead. Space-occupying lesions of water density (kidney cysts) were excluded by preset software thresholds. After that, the percentage of each cortical volume was acquired (Figure 1). There was 1 month interval in evaluating the data of both readers to minimize recall bias.

We calculated the pre-operative SRF for each kidney by multiplying the percentage of either the renal cortical volume (RCV) or DTPA scan by kidney function (CCr and Thai eGFR) as follows:

RCV−SRF=RCV−CT−Volume%∗GFR

DTPA−SRF=DTPA%∗GFR

### Statistical method

Qualitative data were presented as percentage and quantitative data were presented as the mean ± SD. The agreement between RCV-SRF and DTPA-SRF was evaluated by intraclass correlation coefficient (ICC) and Bland–Altman analysis.^12^ The percentage value of systematic error of the Bland–Altman test is about 20%. The relationship between RCV of donated kidney adjusted to recipient body weight and graft function in recipient at 2 weeks and 2 years after transplantation were evaluated by linear regression analysis (Pearson correlation coefficient). Inter-rater reliability was measured by using intraclass correlation analysis. A probability of <0.05 was considered as statistically significant. Statistical analysis was performed using the software SPSS Statistics (v. 22; IBM Corp., Armonk, NY).

## Results

### Patients and demographics

Table 1 provides the baseline characteristics of the 98 patients. 36 patients were males and 62 patients were females with a mean age of 36.3 ± 9 years (range 21–55 years). Interval between CTA of abdominal aorta and DTPA scintigraphy studies ranged from 1 day to 5 months (median = 13 days). The pre-operative kidney function of the donor was as follows: Cr = 1.08±0.2 mg/dl, CCr = 112.9±4.4 mL/min, and Thai eGFR = 103.7±2.7 mL/min/1.73 m^2^.

**Table 1.** Baseline characteristics of the donors

Characteristics	Donors (*N* = 98)
Age (y)	21–55 (36.3)
Sex	
Males	36
Females	62
Pre-operative kidney function	
CCr (mL/min)	76.0–199.0 (112.9 ± 4.4)
Thai eGFR (mL/min/1.73 m^2^)	75.93–205.9 (103.7 ± 2.7)

CCr, creatinine clearance; eGFR, estimated glomerular filtration rate.

### Pre-transplant RCV-SRF and DTPA-SRF

The mean percentage (mean ± SD) of RCV and DTPA scan of the donated kidney were 49.2 ± 2.5 (40.6 to 54) and 50.6 ± 3.2 (42.6 to 57), respectively. The mean percentage of RCV-SRF and DTPA-SRF by CCr were 5538.6 ± 2157.1 and 5699.1 ± 2202.2 and by Thai eGFR were 5092.2 ± 1328.7 and 5256.3 ± 1454.8, respectively. These methods were significantly different (*p* < 0.001).

**Figure 2.**
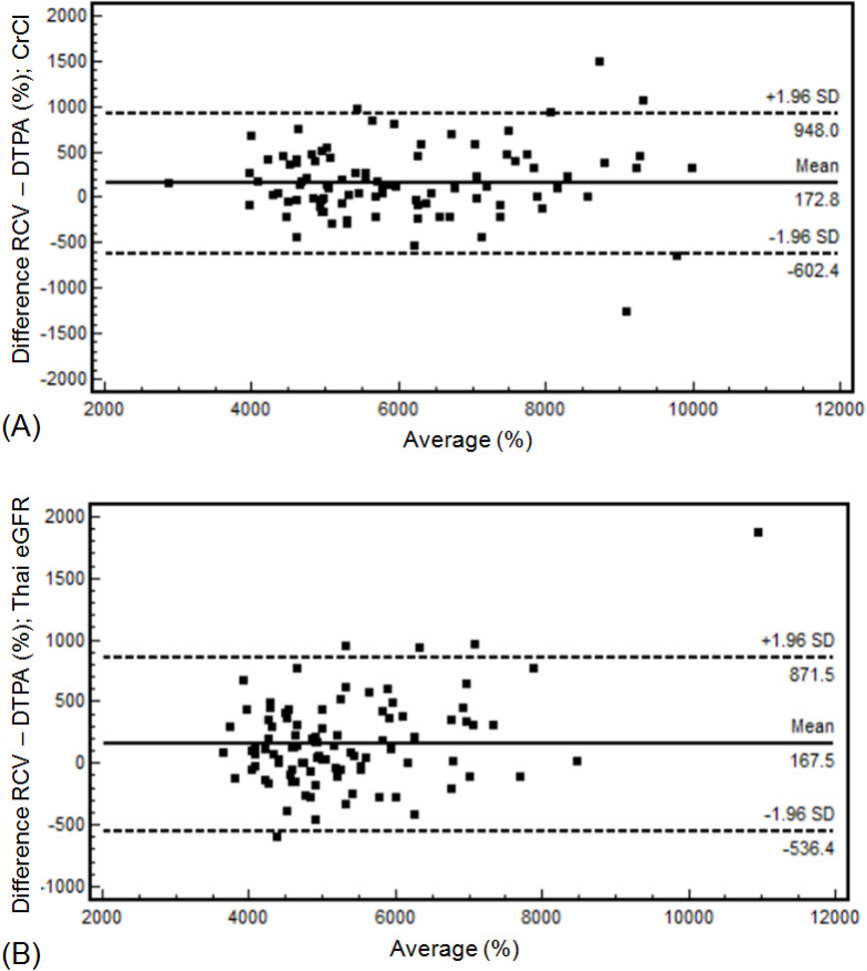
Bland–Altman analysis showed that there was a correlation between pre-donation RCV-SRF and DTPA-SRF; (A) multiplied by CCr (mean = 172.8, 95% limits of agreement from −602.4 to 948); and (B) multiplied by Thai eGFR (mean = 167.5, 95% limits of agreement from −536.4 to 871.5). DTPA, Tc-99m-diethylenetriamine pentacetate; eGFR, estimated glomerular filtration rate; RCV, renal cortical volumes; SRF, split renal function.

There is a strong agreement between pre-transplant RCV-SRF and DTPA-SRF. The ICC was 0.968 (multiplied by CCr) and 0.954 (multiplied by Thai eGFR). These outcomes were affirmed by Bland–Altman plots (Figure 2).

The inter-rater reliability of the two radiologists showed substantial agreement (ICC = 0.97, *p* < 0.001).

### Relationship of RCV of donated kidney adjusted to recipient body weight and graft function in the recipient.

Among all recipients, the mean Cr and CCr at 2 weeks after transplantation were 1.2 ± 0.6 mg/dl and 69.0 ± 21.5 mL/min, respectively. At 2 year follow-up, 25 transplants were excluded because Cr and CCr data were unavailable and 1 recipient died. Overall, data from 73 recipients were analyzed. The mean Cr and CCr at 2 years after transplantation were 1.3 ± 0.5 mg/dl and 72.1 ± 26.6, respectively.

**Figure 3.**
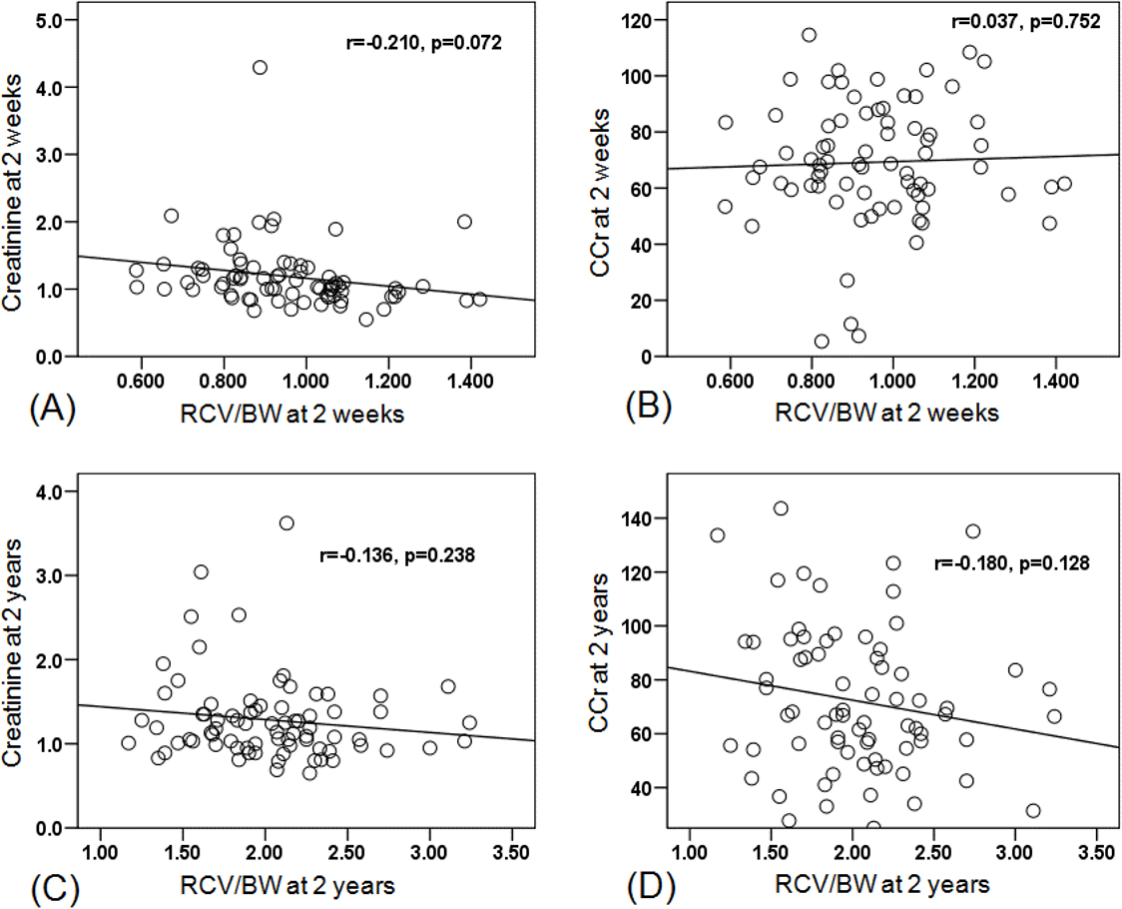
The correlation between RCV/BW and serum Cr or CCr at 2 weeks (A, B) and 2 years (C, D) after transplantation. BW, body weight; RCV, renal cortical volumes.

There were no statistical difference in the correlations between RCV of donated kidney adjusted to recipient body weight (RCV/BW) and serum Cr (*r* = −0.210, *p* = 0.072 and *r* = −0.136 p=0.238) or CCr (*r* = 0.037, *p* = 0.752 and *r* = −0.18, *p* = 0.128) at 2 week and 2 years follow up, respectively (Figure 3). This is inconsistent with prior studies^13–15^ that showed that the prediction of the graft function in the recipients was reproducible.

## Discussion

Evaluation of the SRF in potential living donors before kidney transplantation is requisite to ensure that both the donors and recipients will have sufficient residual kidney function. Moreover, it will provide information to the physicians as which kidney should be used for the kidney transplantation. The more severely affected kidney will usually be used for donation. Many centers continue to use DTPA scintigraphy to estimate SRF.

Our findings showed that there was a strong correlation between CT-based RCV combined with pre-donation renal function and DTPA-measured SRF which was consistent with previous studies.

Halleck et al retrospectively analyzed pre-donation SRF by using CT-measured split cortex volume in 167 LKDs.^10^ The authors showed that CT-based measurement of split cortex volume is capable to replace MAG3 scintigraphy to assess pre-transplant SRF in potential donors.

Wahba et al also reviewed 100 consecutive patients by measuring the percentage of SRF of the preserved kidney by MAG3 scintigraphy and three state-of-the-art CT volumetric techniques (*e.g.* modified ellipsoid volume, smart region of interest volumetry and renal cortex volumetry).^11^ They found that RCV is the most sophisticated volumetric tool that can be used to assess the absolute kidney function.

Furthermore, there is a high reproducibility (ICC = 0.97, *p* < 0.001) between the two radiologists. Therefore, CT-based renal cortex volume appears to be accurate and reproducible to analyze for SRF.

We presumed that RCV/BW was more appropriate than RCV alone in predicting graft function because it probably represents the differential metabolic demand of the recipient. Nonetheless, we found that when we correlated the RCV/BW to kidney function in the recipients either at 2 week or 2 year follow-up, it could not reliably predict the function of the graft in the recipients which was different compared to previous reports.^13–15^ This might be because there are many factors that can affect early postoperative graft function such as delayed graft function, surgical and medical factors.

For long-term follow-up, this study had some limitations. First, the sample size was smaller compared to prior studies. Second, we used the CCr instead of eGFR in assessing the graft function because eGFR is less accurate in patients with renal function that changes rapidly.^16^ However, it should be noted that the CCr can have errors if the patient did not collect the urine specimens accurately and the serum creatinine from the tubular secretion was 15–20% resulting in higher levels of CCr compared to GFR. We need to further evaluate the long-term graft function in a larger sample size and the CCr must be validated by comparing it to the eGFR at the same visit. Third, it is possible that the results are biased because two different CT scanners were used. Last, this is a retrospective study, therefore some information may not be available for this study, particularly in post-transplant recipient.

In conclusion, this study shows that CT-based renal cortex volume combined with pre-operative renal function had valid for estimate the SRF in pre-transplant LKDs, and may therefore obviate the need for additional radioisotopic methods, which might be specially desirable in healthy, young donors. However, CT-based RCV seem not that consistent method to predict the recipient renal function in our population.

There have also been studies using CT-based RCV method for SRF estimation in patients with renal tumors before and after partial nephrectomy. Therefore, it would be interesting to evaluate in other diseases and conditions such as unilateral renovascular disease or obstructive uropathy.
